# Exposure to Concentrated Ambient Particles Does Not Affect Vascular Function in Patients with Coronary Heart Disease

**DOI:** 10.1289/ehp.11016

**Published:** 2008-02-22

**Authors:** Nicholas L. Mills, Simon D. Robinson, Paul H. B. Fokkens, Daan L. A. C. Leseman, Mark R. Miller, David Anderson, Evelyn J. Freney, Mathew R. Heal, Robert J. Donovan, Anders Blomberg, Thomas Sandström, William MacNee, Nicholas A. Boon, Ken Donaldson, David E. Newby, Flemming R. Cassee

**Affiliations:** 1 Centre for Cardiovascular Science, University of Edinburgh, Edinburgh, United Kingdom; 2 National Institute for Public Health and the Environment (RIVM), Bilthoven, Netherlands; 3 ELEGI Colt Laboratory, Centre for Inflammation Research, University of Edinburgh, Edinburgh, United Kingdom; 4 School of Chemistry, University of Edinburgh, Edinburgh, United Kingdom; 5 Department of Respiratory Medicine and Allergy, Umeå University, Umeå, Sweden

**Keywords:** air pollution, blood flow, endothelium, fibrinolysis, inflammation

## Abstract

**Background:**

Exposure to fine particulate air pollution is associated with increased cardiovascular morbidity and mortality. We previously demonstrated that exposure to dilute diesel exhaust causes vascular dysfunction in humans.

**Objectives:**

We conducted a study to determine whether exposure to ambient particulate matter causes vascular dysfunction.

**Methods:**

Twelve male patients with stable coronary heart disease and 12 age-matched volunteers were exposed to concentrated ambient fine and ultrafine particles (CAPs) or filtered air for 2 hr using a randomized, double-blind cross-over study design. We measured peripheral vascular vasomotor and fibrinolytic function, and inflammatory variables—including circulating leukocytes, serum C-reactive protein, and exhaled breath 8-isoprostane and nitrotyrosine—6–8 hr after both exposures.

**Results:**

Particulate concentrations (mean ± SE) in the exposure chamber (190 ± 37 μg/m^3^) were higher than ambient levels (31 ± 8 μg/m^3^) and levels in filtered air (0.5 ± 0.4 μg/m^3^; *p* < 0.001). Chemical analysis of CAPs identified low levels of elemental carbon. Exhaled breath 8-isoprostane concentrations increased after exposure to CAPs (16.9 ± 8.5 vs. 4.9 ± 1.2 pg/mL, *p* < 0.05), but markers of systemic inflammation were largely unchanged. Although there was a dose-dependent increase in blood flow and plasma tissue plasminogen activator release (*p* < 0.001 for all), CAPs exposure had no effect on vascular function in either group.

**Conclusions:**

Despite achieving marked increases in particulate matter, exposure to CAPs—low in combustion-derived particles—did not affect vasomotor or fibrinolytic function in either middle-aged healthy volunteers or patients with coronary heart disease. These findings contrast with previous exposures to dilute diesel exhaust and highlight the importance of particle composition in determining the vascular effects of particulate matter in humans.

Evidence accumulated over more than 50 years of epidemiologic and clinical research has established the adverse effects of air pollution on human health. The London smog of December 1952 caused > 4,000 excess deaths (Ministry of Health 1954); in spite of the dramatic decreases in levels of air pollution that have been achieved since then, the association between air pollution and cardiorespiratory morbidity and mortality persists (Anderson et al*.* 1996; Dockery et al*.* 1993; Pope et al*.* 2002). These associations are strongest for fine particulate matter (PM < 2.5 μm in aerodynamic diameter; PM_2.5_), and the majority of excess deaths are due to cardiovascular events (Pope 2000). Despite the strength of the epidemiologic evidence and the emergence of promising hypotheses, the constituents and biological mechanisms responsible for the cardiovascular effects of air pollution are only beginning to emerge.

Exposure to particulate air pollution has been associated with exercise-induced myocardial ischemia in patients with coronary heart disease (Pekkanen et al*.* 2002) and with triggering acute myocardial infarction (Peters et al*.* 2001a). These findings are limited by exposure misclassification, the effect of potential confounding environmental and social factors, and the lack of mechanistic data (Stone 2004). Controlled exposures of air pollutants can help address these shortcomings by providing a precisely defined exposure in a regulated environment that facilitates investigation with validated biomarkers and surrogate measures of cardiovascular health. Using a carefully characterized exposure system, we previously showed that exposure to dilute diesel exhaust causes lung inflammation (Salvi et al*.* 1999), depletion of airway antioxidant defenses (Behndig et al*.* 2006), and impairment of vascular and fibrinolytic function (Mills et al*.* 2005). Moreover, we recently described ischemic and thrombotic effects in patients with coronary heart disease (Mills et al*.* 2007). Although controlled exposure to diesel exhaust is an excellent model for studying the effects of pure combustion-derived air pollution, we acknowledge that ambient air pollution contains a range of particulate pollutants from a variety of atmospheric sources. In the last few years, technology has been developed that can deliver a continuous flow of air in which the concentration of ambient particulate is increased 10- to 60-fold in real-time (Kim et al*.* 2001; Sioutas et al*.* 1995, 1997). The principle advantage of these concentrators is that they provide real-world inhalation exposures under controlled conditions.

In previous studies, exposure to fine (0.15–2.5 μm in aerodynamic diameter) concentrated ambient particles (CAPs) induced mild pulmonary inflammation in healthy adults (Ghio et al*.* 2000) and altered heart rate variability in the elderly (Devlin et al*.* 2003) and in asthmatic and healthy younger adults (Gong et al*.* 2003). Furthermore, exposure to fine CAPs plus ozone causes peripheral arterial vasoconstriction (Brook et al*.* 2002) and increases in arterial pressure (Urch et al*.* 2005). To date, no studies have addressed the effects of exposure to CAPs in isolation on vascular function, nor have any previous studies assessed the effect of these exposures in an at-risk population of patients with established coronary heart disease. In the present study, we included the ultrafine (particles < 0.15 μm in aerodynamic diameter) fraction of PM. We aimed to assess the effects of a 2-hr exposure to fine and ultrafine CAPs on peripheral vascular vasomotor and fibrinolytic function in patients with stable coronary heart disease and in age-matched healthy controls.

## Materials and Methods

### Subjects

Twelve male patients with stable coronary heart disease and 12 age-matched, male, nonsmoking volunteers participated in these studies, which were performed with the approval of the local research ethics committee, in accordance with the Declaration of Helsinki (World Medical Association 2000), and the written informed consent of all volunteers.

Patients with previous myocardial infarction or stable angina treated by angioplasty and stenting (> 6 months before enrollment) were recruited from the outpatient clinic in the cardiology department at the Royal Infirmary Edinburgh (Edinburgh, Scotland, UK). Patients with symptomatic angina pectoris (Canadian Cardiovascular Society grade ≥ 2; Campeau et al. 1976) or those unable to achieve stage 2 of the Bruce protocol (Bruce 1971) were excluded from the study. Similarly, we excluded any patients with a history of arrhythmia, diabetes mellitus, uncontrolled hypertension, renal or hepatic failure, or unstable coronary disease (acute coronary syndrome or unstable symptoms within 3 months). Matched control subjects were not taking regular medication and had no clinical evidence of atherosclerotic vascular disease, diabetes mellitus, hypertension, or renal or hepatic failure.

In both groups, current smokers and those with asthma, significant occupational exposure to air pollution, or an intercurrent illness were excluded from the study. All subjects had normal lung function as measured by spirometry, and none of them reported symptoms of respiratory tract infection during the study or in the preceding 6 weeks.

### Study design

Subjects attended the clinic on two occasions at least 2 weeks apart and received CAPs or filtered air in a randomized, double-blind cross-over design. Each subject was exposed for 2 hr in a specially built whole-body exposure chamber. During each exposure subjects performed moderate exercise (minute ventilation 25 L/min/m^2^) on a bicycle ergometer that was alternated with rest periods at 15-min intervals.

Based on previous exposure (Mills et al*.* 2005) and systemic inflammatory (Salvi et al*.* 1999) studies, we performed vascular assessments 6–8 hr after CAPs or filtered air exposure. All subjects abstained from alcohol for 24 hr and from food and caffeine-containing drinks for at least 4 hr before each vascular study. Studies were carried out in a quiet, temperature-controlled room maintained at 22–24°C with subjects supine. All subjects remained indoors between the exposure and the vascular assessment to minimize additional exposure to particulate air pollution.

### Concentrated ambient particle exposures and characterization

We used a Versatile Aerosol Concentration Enrichment System (VACES) concentrator (Kim et al*.* 2001), within a Mobile Ambient Particle Concentrator Exposure Laboratory (MAPCEL) to deliver exposures to concentrated CAPs and filtered air. The CAPs were derived from an urban background site outside the Royal Infirmary Edinburgh (Ordnance Survey Grid Reference, NT 289 703) approximately 6 miles from the center of Edinburgh. A bus route passed adjacent to the MAPCEL, and an arterial city route was located a few hundred meters away. To maximize differences in exposure and minimize the influence of ambient pollution, most CAPs exposures were conducted when ambient pollution levels were high. On the day of the first exposure, the MAPCEL technicians made a considered judgment as to whether atmospheric conditions were better suited to conducting a CAPs or filtered air exposure, and subjects were randomized accordingly. The study investigators and staff remained blinded to exposure allocation throughout the study.

**Figure 1:**
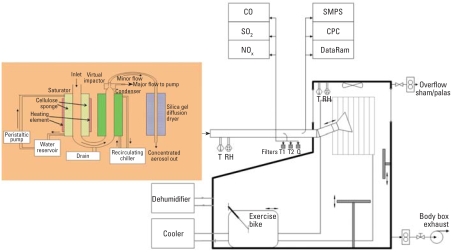
A schematic diagram of the VACES used to deliver CAPs and filtered air to human subjects. Abbreviations: CO, carbon monoxide; CPC, condensation particle counter; NOx, nitrogen oxides; Q, quartz filter; RH, relative humidity; SMPS, scanning mobility particle sizer; SO2, sulfur dioxide; T, temperature; T1 and T2, teflon filters. See “Materials and Methods” for details.

A schematic diagram of the MAPCEL and VACES used to deliver CAPs and filtered air to human subjects is given in Figure 1. Incoming ambient air (500 L/min) is saturated with water vapor to increase the size of PM_2.5_ before the air is passed through five parallel virtual impactors, each operating at 100 L/min. This increase in size and therefore mass ensures that particles have sufficient momentum to pass through the impactors exiting in the minor flow (5 L/min) in which the particle concentration is enriched by a factor of 10- to 20-fold. Of note, particles > 3 μm are lost by impaction on the walls of the inlet and saturator and do not pass through this system. Similarly, particles < 15–20 nm do not grow in the condensation unit and are lost to the exhaust in the major flow from the impactors. The outward minor flow from the five impactors (25 L/min) is desaturated by silica gel dryers to restore the particles to their original size and diluted with filtered air before delivery into the human exposure chamber (50 L/min).

We continuously monitored the air in the exposure chamber for temperature, humidity, nitrogen oxides [chemiluminescence NO-NO_2_- NO_x_ analyzer; Thermo Environmental Instruments (TEI), Franklin, MA, USA], carbon monoxide (gas filter correlation CO analyzer; TEI) sulfur dioxide (pulsed fluorescence SO_2_ analyzer; TEI), and ozone (absorption ozone analyzers model 8810; Monitor Labs, San Diego, CA, USA). We determined particle number using a condensation particle counter (Model 3022A; Thermo Systems Incorporated, St. Paul, MN, USA). Particle mass was continuously monitored using a DataRam nephelometer (Measuring Instruments for the Environment Corporation, Bedford, MA, USA) with the precise mass determined by gravimetric filter measurements (Teflon 2.0 μm, 4.7 mm; PALL Life Sciences, Ann Arbor, MI, USA). The concentration of CAPs was not standardized for all subjects because exposures were dependent on ambient PM levels on the day of the study. We aimed to deliver approximately 200 μg/m^3^ to allow comparison with previously published studies (Mills et al*.* 2005).

We used an aerosol time-of-flight mass spectrometer (ATOFMS; TSI) to characterize single particles sampled during the CAPS exposures. The operation of the ATOFMS instrument has been described in detail elsewhere (Toner et al*.* 2006), and detailed results are presented elsewhere (Freney et al*.* 2006).

### Vascular studies

All subjects underwent brachial artery cannulation with a 27-gauge steel needle under controlled conditions. After a 30-min baseline saline infusion, acetylcholine at 5, 10, and 20 μg/min [endothelium-dependent vasodilator that does not release tissue plasminogen activator (t-PA); Merck Biosciences, Läufelfingen, Switzerland], bradykinin at 100, 300, and 1,000 pmol/min (endothelium-dependent vasodilator that releases t-PA; Merck Biosciences), and sodium nitroprusside at 2, 4, and 8 μg/min (endothelium-independent vasodilator that does not release t-PA; David Bull Laboratories, Warwick, UK) were infused for 6 min at each dose. The three vasodilators were separated by 20-min saline infusions and given in a randomized order. We measured forearm blood flow in the infused and noninfused arms by venous occlusion plethysmography using mercury-in-silastic strain gauges as described previously (Newby et al*.* 1999). We monitored supine heart rate and blood pressure in the noninfused arm at intervals throughout each study using a semiautomated, noninvasive oscillometric sphygmomanometer. For each subject, venous cannulae (17 gauge) were inserted into large subcutaneous veins of the ante-cubital fossae of both arms. Blood (10 mL) was withdrawn simultaneously from each arm at baseline and during the infusion of each dose of bradykinin, and collected into acidified buffered citrate (Stabilyte tubes; Biopool International, Ventura, CA, USA) for t-PA assays, and citrate (BD Vacutainer; Franklin Lakes, NJ, USA) for plasminogen activator inhibitor type 1 (PAI-1) assays. Samples were kept on ice and then centrifuged at 2,000 × *g* for 30 min at 4°C. Platelet-free plasma was decanted and stored at −80°C before assays. We determined plasma t-PA and PAI-1 antigen concentrations by ELISA (enzyme-linked immunosorbant assay kits: TintElize t-PA, Biopool International; Coaliza PAI-1, Chromogenix AB, Mölndal, Sweden). Intraassay coefficients of variation were 7.0% and 5.5% for t-PA and PAI-1 antigen, with interassay coefficients of variability of 4.0% and 7.3%, respectively. We determined hematocrit by capillary tube centrifugation at baseline and during infusion of bradykinin 1,000 pmol/min.

Blood samples were taken immediately before exposure and at 6 and 24 hr after the exposure and analyzed for total cells, differential count, and platelets using an autoanalyzer. We measured serum C-reactive protein (CRP) concentrations using a highly sensitive immunonephelometric assay (Behring BN II nephelometer, Dade-Behring, Marburg, Germany). Intraassay and interassay coefficients of variability were 3.0% and 4.6%, respectively.

### Exhaled breath condensate

In a pilot study, exhaled breath condensate (EBC) was collected using a Jaeger Ecoscreen (VIASYS Healthcare, Hoechberg, Germany) immediately before exposure and at 6 and 24 hr after exposures in eight healthy volunteers. Subjects rinsed their mouths with water immediately before the collection. We collected EBC during a 10-min period of normal tidal breathing through the mouthpiece with a nose clip in place. Samples were placed on ice immediately after collection and aliquoted for storage at −80°C within 30 min. We measured 8-isoprostane and nitrotyrosine in EBC using commercially available ELISA kits (Quantikine; R&D Systems, Minneapolis, MN, USA). The intraassay and interassay coefficients of variation for the assays were 5% and 6%, respectively.

### Data analysis and statistics

Plethysmographic data were analyzed as described previously (Newby et al*.* 1999). Estimated net release of t-PA antigen was defined as the product of the infused forearm plasma flow (based on the mean hematocrit and the infused forearm blood flow) and the concentration difference between the infused and noninfused arms (Newby et al*.* 1997; Oliver et al*.* 2005). We based the sample size of 12 subjects in each group on power calculations derived from previous studies to give 90% power of detection for a 17% difference in mean t-PA release and a 22% difference in forearm blood flow at a significance level of 5% (Newby et al. 1997, 1999). Continuous variables are reported as mean ± SE. Statistical analyses were performed with GraphPad Prism (GraphPad Software, San Diego, CA, USA) using analysis of variance (ANOVA) with repeated measures, Pearson’s correlation, two-tailed Student’s *t*-test, or Fisher’s exact test where appropriate. The area under the curve was calculated for the estimated net release of t-PA during the forearm study period. Statistical significance was taken at *p* < 0.05.

## Results

All patients had proven coronary heart disease with a previous myocardial infarction or stable angina treated by angioplasty and stenting (> 6 months before enrollment) and were receiving standard secondary preventive therapy (Table 1). Subjects tolerated the exposures well and did not report any symptoms during the exposure or in the 24 hr after each exposure. Patients were well matched for age and blood pressure but had greater body mass index than the healthy controls (*p* < 0.05).

### Exposures

Ambient PM concentrations during the exposure period were variable (range, 3–174 μg/m^3^) across the 3-month duration of the study, with an average ambient concentration of 20 ± 4 μg/m^3^. Although we were able to enrich the concentration of PM 6- to 8-fold, variation in ambient levels resulted in a wide range of PM exposures between 50 and 682 μg/m^3^. Average PM concentrations in the CAPs exposures (190 ± 37 μg/m^3^) were greater than those for ambient levels and filtered air (31 ± 8 μg/m^3^ and 0.5 ± 0.4 μg/m^3^, respectively; *p* < 0.001). We found no significant differences between concentrations of gaseous copollutants between the filtered air or CAPs exposures (Table 2). There were, however, differences in humidity and temperature in the chamber between the CAPs and filtered air exposures that occurred as a result of the saturation and dilution phases of the enrichment process.

**Figure 2:**
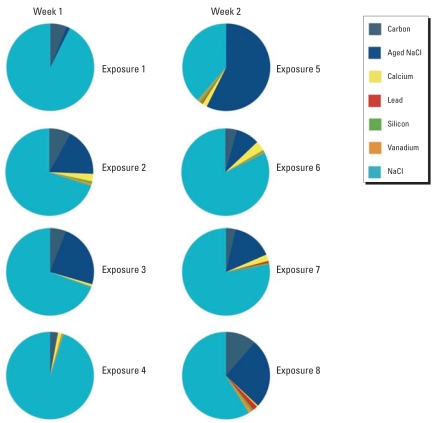
ATOFMS analysis of ambient particulate during each 2-hr exposure in a representative 2-week period. Data are presented as the percentage of particles analyzed containing calcium, elemental carbon, silicon, vanadium, and sodium chloride or aged NaCl. The ATOFMS detected NaCl as the primary chemical constituent of ambient aerosol, and only low levels of elemental carbon.

Using the ATOFMS, elemental analysis of ambient PM was acquired during each 2-hr exposure for a representative 2-week period of the study. The ATOFMS detected low levels of carbon (5.3 ± 1.2% of all analysed particles) and sodium chloride (91.7 ± 6.5%) as the primary chemical constituents of ambient aerosol (Figure 2).

### Vascular function

**Figure 3:**
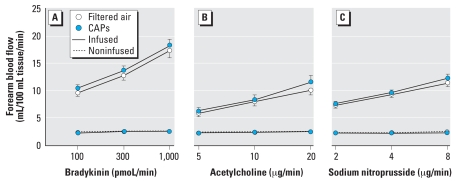
Infused and noninfused forearm blood flow in all subjects (*n* = 24) after CAPs and filtered air exposures during intrabrachial infusion of bradykinin (*A*), acetylcholine (*B*), and sodium nitroprusside (*C*). Values shown are mean ± SE. For all dose responses in the infused arm, *p* < 0.0001. *p*-Values for CAPs versus filtered air are as follows: bradykinin, *p* = 0.20; acetylcholine, *p* = 0.17; and sodium nitroprusside, *p* = 0.14.

We observed no differences in resting heart rate, blood pressure, or baseline forearm blood flow after exposure to CAPs or filtered air in either cohort (Table 3). Bradykinin, acetylcholine, and sodium nitroprusside caused dose-dependent increases in forearm blood flow following both air and CAPs exposure (*p* < 0.0001); however, this increase in blood flow was not affected by exposure to CAPs or filtered air in either patients or controls (Figure 3). Bradykinin caused a dose-dependent increase in plasma t-PA antigen concentrations (*p* < 0.0001) that was similarly unaffected by exposure (Table 4). We found no correlation between peak forearm blood flow and particle mass or particle number in the CAPs exposure for any of the vasodilators infused.

### Markers of oxidative stress and inflammation

**Figure 4:**
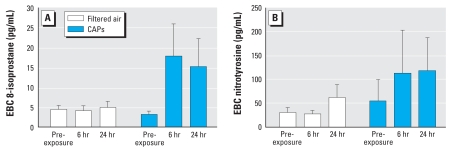
EBC levels of 8-isoprostane (*A*) and nitrotyrosine (*B*) in healthy volunteers (*n* = 8) at 6 and 24 hr after exposure to CAPs or filtered air. (*A*) EBC levels of 8-isoprostane increased at both time points after CAPs exposure (ANOVA, *p* = 0.04, CAPS compared to filtered air). (*B*) Nitrotyrosine concentrations did not change significantly (ANOVA, *p* = 0.21). Values shown are mean ± SE.

EBC levels of 8-isoprostane, produced by oxidation of tissue phospholipids, increased 6 and 24 hr after CAPs exposure compared with exposure to filtered air (ANOVA, *p* = 0.04; Figure 4), whereas levels of nitrotyrosine, an indicator of nitrosative stress, did not significantly differ between exposures. In all subjects, we observed a small increase in the number of circulating platelets (234 ± 8 vs. 225 ± 8 × 10^9^/L; *p* = 0.007) at 2 hr and monocytes (0.58 ± 0.03 vs. 0.53 ± 0.03 × 10^9^/L; *p* = 0.03) at 6 hr after exposure to CAPs compared with exposure to filtered air (Table 5). There was, however, no clear evidence of a systemic inflammatory response to CAPs exposure: Total leukocyte, neutrophil, and lymphocyte counts or serum CRP concentrations were unaltered by CAPs or air exposure at any time point.

## Discussion

Exposure to concentrated ambient PM in a typical urban environment for 2 hr did not affect vascular vasomotor or endogenous fibrinolytic function in either middle-aged healthy volunteers or patients with coronary heart disease. The ambient PM was generally low in elemental carbon, indicating that combustion sources were not a major source of ambient particulate. However, inhalation of CAPs caused mild pulmonary oxidative stress, which did not result in a significant systemic inflammatory response. Exposure to particulate air pollution low in combustion component, at concentrations 3- to 5-fold higher than the U.S. Environmental Protection Agency (EPA) National Ambient Air Quality Standards (NAAQS; U.S. EPA 2008), is unlikely to cause adverse vascular effects capable of triggering an acute coronary event.

These findings contrast with those from our previous studies in which we reported impairment of vascular function in both healthy volunteers and patients with coronary heart disease after a 1-hr exposure to dilute diesel exhaust (Mills et al*.* 2005; Törnqvist et al*.* 2007). This apparent discrepancy requires further discussion, with differences in particle number, particle composition, and the presence of gaseous copollutants between these exposures potentially responsible.

### Ambient particle and diesel exhaust exposures

We were able to increase the concentration of ambient PM 6- to 8-fold to deliver exposures between 50 and 682 μg/m^3^. The majority of exposures were substantially higher than the U.S. EPA standard of 35 μg/m^3^ for daily PM_2.5_ (U.S. EPA 2008). Exposure to 190 μg/m^3^ for 2 hr is roughly comparable to a 1-hr exposure to the 300 μg/m^3^ of diesel exhaust particulate delivered in previous studies. We are confident that these estimates of exposure are accurate because we determined the precise mass by gravimetric filter measurements. It is possible that vascular dysfunction only occurred in subjects exposed to the highest concentrations of CAPs and that a significant overall effect of exposure to CAPs on vascular function was masked by those volunteers receiving only modest particle exposures. This seems unlikely, however, given we found no relationship between particle number or mass and vasodilatation to any of the endothelial-dependent or endothelial-independent agonists infused.

Although we delivered a similar mass of PM in the present study and previous exposures to diesel exhaust, there were important differences in particle size and therefore particle number concentration between the exposures. In dilute diesel exhaust, the PM consists of pure combustion-derived carbon nanoparticulate. These particles typically range from 20 to 120 nm, with a count median diameter of 54 nm (geometric SD = 1.7 nm) (Mills et al*.* 2007). The VACES system does not include particles < 15 nm in the exposure atmosphere, and the mean particle size in a typical CAPs exposure was 1.23 ± 0.4 μm in diameter. Therefore, the total number of suspended particles in dilute diesel exhaust was 10-fold more than in the CAPs exposure. Toxicologic studies have determined that the adverse oxidative and pro-inflammatory effects of particles are in part determined by surface area (Duffin et al*.* 2007; Oberdorster et al*.* 2005; Warheit et al*.* 2007). It follows that smaller particles exert a greater effect per unit mass than larger particles of similar toxicity. It is therefore possible that the lack of adverse vascular effects after exposure to CAPs reflects the much lower number of nanosized particles in the present study.

Diesel exhaust is a complex mixture of gases and particles, and although we hypothesize that our previous findings are principally due to an effect of combustion-derived particles, it is not possible to definitively exclude a nonparticulate cause of the adverse vascular effects. Potentially important copollutants produced in the combustion of diesel oil include NO_x_ and CO. One of the advantages of the VACES system is that it allows us to study the effects of particulates alone, and the enrichment process does not alter the concentration of gaseous pollutants. Concentrations of NO_2_ and CO were low and easily within the recommended NAAQS (U.S. EPA 2008) in our present study. In epidemiologic studies, ambient NO_2_ or CO have been primarily considered surrogates for traffic-derived pollution (Brunekreef and Holgate 2002), but it is possible that these and other organic gaseous pollutants in diesel exhaust exert a synergistic effect with the particulates.

As an important source of combustion-derived particulate, diesel exhaust is strongly implicated in the observed adverse effects of air pollution. A variable proportion of urban PM is attributable to combustion-derived nanoparticles from traffic, ranging from 20% at remote monitoring sites (Lanki et al*.* 2006) up to 70% in a road tunnel (Geller et al*.* 2005). In Edinburgh CAPs, only 5% of particles analyzed contained elemental carbon, and the principle constituent was pure, mixed, or reacted sea salts (90%). This is not surprising given Edinburgh’s maritime climate. Air-mass source attribution plots for the 5 days before arrival at the sampling location were calculated for each of the exposure periods using a U.K. Meterological Office model (Manning et al. 2003). In all cases, air clearly originated either predominantly from the Atlantic or the Arctic, with very little contribution from air passing over land apart from final arrival over central Scotland (Freney et al*.* 2006). The proportion of airborne PM derived from remote combustion sources is likely to be low. It is likely that the absence of any detrimental vascular effects in the present study, in part, reflects the composition of Edinburgh CAPs, which are likely to be of very low toxicity. Although epidemiologic studies propose that PM mass is the metric most strongly associated with adverse events, our studies suggest that composition strongly affects potency and is likely to be the main effector of outcome.

### Air pollution, oxidative stress, and inflammation

A substantial body of evidence supports a role for oxidative stress in determining the toxicity of ambient pollution (Donaldson et al*.* 2005a) and in the pro-inflammatory effects of combustion-derived particles (Donaldson et al*.* 2005b; Nemmar et al*.* 2003). Reactive oxidant species arise not only from the redox potential of the pollutants themselves but also from the activation of alveolar epithelial cells or resident macrophages and the recruitment and activation of circulating neutrophils. In the present study, we used an emerging noninvasive method of assessing pulmonary oxidative stress through collection of EBC (Montuschi et al*.* 1999). In the preliminary studies, we found an increase in EBC 8-isoprostane in normal subjects, suggesting that inhaled ambient particles exert a pro-oxidant effect in the airways. This is the first time, to our knowledge, this technique has been used to assess the effects of exposure to air pollutants. If these findings can be replicated and extended in a larger study, breath condensate measures may become a useful biomarker of PM exposure.

In panel and population studies, PM exposure is associated with evidence of an acute-phase response with increased CRP (Peters et al*.* 2001b) and plasma fibrinogen (Pekkanen et al*.* 2000; Schwartz 2001), enhanced plasma viscosity (Peters et al*.* 1997), and altered hematologic indices (Seaton et al*.* 1999). In animal studies, there are similar reports, with increased fibrinogen in the blood of PM-exposed hypertensive rats (Cassee et al*.* 2002, 2005) and normal rats exposed to ultrafine carbon particles (Elder et al*.* 2004). In recent years CRP has come to prominence as a biological marker of atherosclerosis, with serum CRP concentrations, even at very low levels, predicting the risk of acute myocardial infarction or stroke in apparently healthy individuals (Ridker et al. 1997). Serum CRP concentrations reflect the burden of vascular inflammation; however, in the presence of an intercurrent illness or an exogenous inflammatory stimulus, serum CRP concentrations increase rapidly as part of the acute-phase response. We did not find a consistent systemic inflammatory signal after CAPs exposure: there were no changes in the numbers of circulating neutrophils, lymphocytes, or total leukocytes, even up to 24 hr. Similarly, we did not find an increase in serum CRP concentrations, suggesting that a 2-hr exposure to CAPs may not be sufficient to induce a sustained systemic inflammatory response.

The number of circulating platelets increased 2 hr after CAPs exposure, and a similar transient effect on circulating monocytes was present at 6 hr. Whether these small changes are likely to increase cardiovascular risk is questionable. The cellular mechanisms of atherosclerosis are complex, but adhesion of platelets and monocytes to the damaged arterial wall occurs early in response to vascular injury (Fuster et al*.* 1992). Activated platelets deposit at sites of plaque rupture and may precipitate coronary artery occlusion. Platelet–monocyte aggregates are increased in cigarette smokers (Harding et al*.* 2004) and in patients with unstable angina (Sarma et al*.* 2002), suggesting that leukocyte–platelet interactions may contribute to, or be a marker of, atheromatous plaque instability. Although we did not measure markers of platelet or monocyte activation in our study, Frampton et al*.* 2006 recently reported that inhalation of carbon ultrafine particles alters leukocyte expression of adhesion molecules in peripheral blood.

### Study limitations

Using a robust randomized, double-blind sham-exposure study design, we have assessed the effect of CAPs on two complementary aspects of vascular function. Impaired vasodilation and fibrinolytic function in the forearm vascular bed have previously been shown to independently predict adverse outcomes in patients with coronary heart disease (Heitzer et al*.* 2001; Robinson et al*.* 2007), and therefore we believe this model is a reasonable surrogate for cardiovascular health. Furthermore, these studies were conducted in a highly relevant population of patients with existing coronary heart disease who are likely to be susceptible to the adverse effects of air pollution and for whom a better understanding of the effects of exposure to particulate air pollution is of clinical importance. The findings are clear: A 2-hr exposure to increased concentrations of ambient PM in an urban setting does not have significant effects on systemic vascular function.

Our findings are consistent with the only previous study of the cardiovascular effects of CAPs in humans (Brook et al. 2002); in that study, the authors assessed the effect of a 2-hr exposure to CAPs using a concentrator technology that does not enrich ultrafine particles (Sioutas et al. 1997). Flow-mediated dilation assessed by ultrasound of the brachial artery was used to determine conduit vessel function rather than venous occlusion plethysmography that measures peripheral resistance vessel function. Although CAPs exposure did not affect endothelium-dependent or independent vasodilatation in Brook et al.’s study, it did induce vasoconstriction of the brachial artery. It is possible that our CAPs exposures would have induced similar effects on conduit arterial tone because this may not be reflected in changes in basal forearm blood flow measured by venous occlusion plethysmography.

It is not possible to generalize our findings or state that exposure to PM is not likely to exert harmful vascular effects in other urban settings. This is primarily because the maritime climate and location of the MAPCEL resulted in an exposure to low levels of combustion component. The concentrator technology relies on environmental conditions on the study date to ensure that a relevant exposure can be delivered, and therefore we were not able to give a prespecified concentration of ambient particulate. While we delivered almost 200 μg/m^3^ CAPS, it is possible that higher concentrations even of low toxicity CAPs would have had more potent adverse effects. Further studies in different cities and city locations are clearly warranted.

## Conclusions

Despite achieving substantial increases in ambient PM concentrations in an urban setting, exposure to ambient particulate air pollution for 2 hr had no effect on vascular vasomotor or endogenous fibrinolytic function in either healthy middle-aged volunteers or patients with established coronary heart disease. These findings suggest that exposure to PM that is low in combustion component is unlikely to exert significant vascular effects capable of triggering an acute coronary event.

## Figures and Tables

**Table 1:** Baseline characteristics of coronary heart disease patients and controls.

Characteristic	Controls	Patients	*p*-Value
Age (years)	54 ± 2	59 ± 2	0.12
Height (cm)	181 ± 1	176 ± 2	0.03
Weight (kg)	81 ± 3	87 ± 3	0.14
Body mass index (m^2^/kg)	25 ± 1	28 ± 1	0.02
FEV_1_ (L)	3.4 ± 0.1	3.0 ± 0.2	0.04
FVC (L)	4.6 ± 0.2	4.2 ± 0.2	0.07
FEV_1_/FVC	74 ± 1	72 ± 2	0.32
Systolic blood pressure (mmHg)	135 ± 5	138 ± 6	0.69
Diastolic blood pressure (mmHg)	77 ± 3	80 ± 3	0.50
Heart rate (beats per minute)	63 ± 3	54 ± 1	0.006
Previous myocardial infarction	0	7	0.005
Previous PTCA	0	7	0.005
Previous CABG	0	1	0.5
Current smokers	0	0	1.0
Ex-smokers	3	5	0.67
Nonsmokers	9	7	0.67
Hypertension	0	4	0.09
Hypercholesterolemia	0	12	< 0.001
Drugs			
Aspirin	0	12	< 0.001
Statins	0	12	< 0.001
Beta blockers	0	11	< 0.001
ACE inhibitors/ARB^a^	0	7	0.001

Abbreviations: ACE, angiotensin converting enzyme; ARB, angiotensin receptor blocker; CABG, coronary artery bypass grafting; FEV_1_, forced expiratory volume in 1 sec; FVC, forced vital capacity; PTCA, percutaneous transluminal coronary arteriography. Values shown are mean ± SE or number.

a ACE inhibitor therapy was withdrawn 7 days before each vascular study; all other regular medications were continued throughout the study.

**Table 2:** Exposure variables.

Variable	Air	CAPs	*p*-Value
PM (Teflon filter) (μg/m^3^)	—	178 ± 46	< 0.001
PM (quartz filter) (μg/m^3^)	—	162 ± 22	< 0.001
PM (DataRam) (μg/m^3^)	0.5 ± 0.4	190 ± 37	< 0.001
Ambient PM (DataRam) (μg/m^3^)	9 ± 1	31 ± 8	0.01
Particle number (1,000 × number/cm^3^)	—	99.4 ± 9.5	< 0.001
Ozone (ppb)	6.0 ± 1.3	5.0 ± 1.2	0.52
Carbon monoxide (ppb)	27 ± 1	24 ± 3	0.40
Sulfur dioxide (ppb)	0.13 ± 0.07	0.13 ± 0.07	1.00
Nitric oxide (ppb)	4.5 ± 0.3	4.6 ±1.0	0.93
Nitrogen dioxide (ppb)	5.9 ± 0.7	5.1 ± 1.0	0.52
NO_x_ (NO + NO_2_) (ppb)	6.3 ± 0.7	7.2 ± 1.7	0.60
Temperature (°C)	21.1 ± 0.2	20.1 ± 0.2	0.004
Relative humidity (%)	28 ± 2	57 ± 2	< 0.001

Values shown are mean ± SE or number (*n* = 24).

**Table 3:** Hemodynamic variables 6 hr after exposure in all subjects.

	Exposure		
Variable	Air	CAPs	*p*-Value
Heart rate (beats per minute)	58 ± 2	58 ± 1	0.94
Systolic blood pressure (mmHg)	135 ± 4	135 ± 4	0.51
Diastolic blood pressure (mmHg)	78 ± 2	78 ± 2	0.96
Mean arterial pressure (mmHg)	97 ± 2	97 ± 3	0.70
Infused FBF (mL/100 mL tissue/min)	2.6 ± 0.3	2.5 ± 0.2	0.57
Noninfused FBF (mL/100 mL tissue/min)	2.7 ± 0.2	2.3 ± 0.2	0.16

FBF, forearm blood flow. Values shown are mean ± SE (*n* = 24), two-tailed paired *t*-test.

**Table 4:** Plasma t-PA antigen concentrations after exposure to filtered air or CAPs.

	Air				CAPs			
Bradykinin (pmol/min)	0	100	300	1,000	0	100	300	1,000
tPA (ng/mL)								
Noninfused arm	7.6 ± 0.5	7.4 ± 0.5	7.9 ± 0.5	8.5 ± 0.5	7.2 ± 0.5	7.3 ± 0.6	7.7 ± 0.5	8.6 ± 0.5
Infused arm	7.3 ± 0.5	8.8 ± 0.5	9.7 ± 0.6	14.3 ± 1.2*	7.3 ± 0.6	8.6 ± 0.6	9.5 ± 0.7	14.2 ± 1.1*
Difference	−0.4 ± 0.2	1.4 ± 0.4	1.9 ± 0.4	5.8 ± 1.1*	0.1 ± 0.2	1.3 ± 0.4	1.7 ± 0.4	5.7 ± 1.0*
Net t-PA release (ng/100 mL tissue/min)	−1 ± 1	11 ± 3	20 ± 5	90 ± 18*	0 ± 1	12 ± 4	24 ± 6	104 ± 19*

Values shown are mean ± SE (*n* = 24).

* *p* < 0.0001 (ANOVA; dose response).

**Table 5:** Systemic effects of exposure to filtered air or CAPs.

	Air				CAPs				
	Preexposure	2 hr	6 hr	24 hr	Preexposure	2 hr	6 hr	24 hr	ANOVA
Leukocytes (× 10^9^ cells/L)	5.8 ± 0.2	5.8 ± 0.2	6.4 ± 0.2	5.6 ± 0.2	5.8 ± 0.2	6.1 ± 0.2	6.4 ± 0.2	5.5 ± 0.2	0.31
Neutrophils (× 10^9^ cells/L)	3.2 ± 0.1	3.5 ± 0.1	3.9 ± 0.2	3.3 ± 0.1	3.3 ± 0.1	3.7 ± 0.2	3.9 ± 0.2	3.2 ± 0.2	0.58
Lymphocytes (× 10^9^ cells/L)	1.7 ± 0.1	1.5 ± 0.1	1.7 ± 0.1	1.5 ± 0.2	1.6 ± 0.1	1.6 ± 0.1	1.8 ± 0.1	1.5 ± 0.1	0.47
Monocytes (× 10^9^ cells/L)	0.57 ± 0.03	0.50 ± 0.03	0.53 ± 0.03	0.49 ± 0.03	0.55 ± 0.04	0.55 ± 0.04	0.58 ± 0.04*	0.50 ± 0.03	0.02
Platelets (× 10^9^ cells/L)	221 ± 7	225 ± 8	218 ± 7	223 ± 7	224 ± 8	234 ± 8*	217 ± 6	228 ± 8	0.04
CRP (mg/L)	1.2 ± 0.2	—	1.1 ± 0.2	1.3 ± 0.2	1.3 ± 0.2	—	1.2 ± 0.2	1.2 ± 0.2	0.72
PAI-1 antigen (ng/mL)	57 ± 5	33 ± 3	19 ± 2	48 ± 5	63 ± 5	34 ± 3	19 ± 2	51 ± 4	0.30
t-PA antigen (ng/mL)	10.1 ± 0.6	10.6 ± 0.7	7.2 ± 0.5	10.6 ± 0.7	10.2 ± 0.8	10.5 ± 0.7	7.2 ± 0.5	10.9 ± 0.7	0.86

Values shown are mean ± SE (*n* = 24). Repeated-measure ANOVA CAPs versus filtered air.

* *p* < 0.05; Student’s *t*-test, CAPs versus filtered air (time point).
